# Activation of (pro)renin by (pro)renin receptor in extracellular vesicles from osteoclasts

**DOI:** 10.1038/s41598-021-88665-y

**Published:** 2021-04-28

**Authors:** Jonathan B. Murray, Christy Mikhael, Guanghong Han, Lorraine Perciliano de Faria, Wellington J. Rody, L. Shannon Holliday

**Affiliations:** 1 Department of Orthodontics, University of Florida College of Dentistry, Gainesville, FL 32610 USA; 2 Department of Oral Geriatrics, Hospital of Stomatology, Jilin University, Changchun, 130021 People’s Republic of China; 3 Department of Biomaterials and Oral Biology, School of Dentistry, University of São Paulo, São Paulo, 01000 Brazil; 4 Department of Orthodontics and Pediatric Dentistry, Stony Brook University School of Dental Medicine, Stony Brook, NY 11794 USA; 5 Department of Anatomy & Cell Biology, University of Florida College of Medicine, Gainesville, FL 23610 USA; 6 Department of Orthodontics, University of Florida College of Dentistry, 1600 SW Archer Road, CB 1000444, Gainesville, FL 23610 USA

**Keywords:** Biochemistry, Cell biology

## Abstract

The (pro)renin receptor (PRR) is a multifunctional integral membrane protein that serves as a component of the vacuolar H^+^-ATPase (V-ATPase) and also activates (pro)renin. We recently showed that full-length PRR, found as part of a V-ATPase sub-complex, is abundant in extracellular vesicles shed by osteoclasts. Here, we tested whether these extracellular vesicles stimulate (pro)renin. Extracellular vesicles isolated from the conditioned media of RAW 264.7 osteoclast-like cells or primary osteoclasts were characterized and counted by nanoparticle tracking. Immunoblotting confirmed that full-length PRR was present. Extracellular vesicles from osteoclasts dose-dependently stimulated (pro)renin activity, while extracellular vesicles from 4T1 cancer cells, in which we did not detect PRR, did not activate (pro)renin. To confirm that the ability of extracellular vesicles from osteoclasts to stimulate (pro)renin activity was due to the PRR, the “handle region peptide” from the PRR, a competitive inhibitor of PRR activity, was tested. It dose-dependently blocked the ability of extracellular vesicles to stimulate the enzymatic activity of (pro)renin. In summary, the PRR, an abundant component of extracellular vesicles shed by osteoclasts, stimulates (pro)renin activity. This represents a novel mechanism by which extracellular vesicles can function in intercellular regulation, with direct implications for bone biology.

## Introduction

Local renin/angiotensin signaling (RAS) is involved in regulating bone remodeling and is associated with osteoporosis^1–4^. Elements of the RAS system are locally synthesized in bone tissue^2^. Both osteoclasts, bone-degrading hematopoietic cells, and osteoblasts, bone forming mesenchymal cells, respond to RAS through angiotensin (Ang) receptors^5^. Inhibition of RAS increased bone mineral density, and increases in RAS signaling triggered bone loss^4,6–8^. In addition, polymorphisms in angiotensin converting enzyme (ACE) are associated with risk of osteoporotic fractures^3^.

RAS is best known for its role in regulating blood pressure^9^. This involves the “circulating” RAS where renin or activated (pro)renin, produced by the kidney, acts on liver-derived angiotensinogen to generate angiotensin (Ang) I. Ang II, which stimulate Ang receptors, is then produced by cleavage of Ang I by ACE. In addition to the circulating RAS, there are tissue-based mechanisms for Ang peptide formation. The key feature of “local” RAS is the local synthesis of important components of RAS^2,8,10^. These are ACE and Ang II type 1 (AT1) and AT2 receptors. Other components may include local synthesis of angiotensinogen and the (pro)renin receptor (PRR, also known as ATP6AP2), which activates (pro)renin to cleave angiotensinogen.

We reported proteomic analysis of extracellular vesicles (EVs) shed by osteoclasts^11^. EVs are 30–150 nm in diameter vesicles that are released from cells either as multivesicular bodies fuse with the plasma membrane (exosomes), or by direct budding from the plasma membrane (microvesicles)^12^. Accumulating evidence indicates that EVs from osteoclasts may be crucial signaling molecules. Regulatory EVs containing receptor activator of nuclear factor kappa B (RANK) were identified by our group^13^. These were later shown to be “coupling factors” that stimulate osteoblasts to form bone to compensate for the removal of old bone by osteoclasts and thereby help maintain bone strength^14^. In our proteomic analysis of EVs from osteoclasts, we found that PRR was abundant^11^. The PRR was full length, and was associated with a sub-complex of vacuolar H^+^-ATPase (V-ATPase) subunits^11^.

PRR is a multifunctional protein^15^. While it was long known to be an associated with V-ATPase^16^, and was thought to be an accessory protein required for the assembly of V-ATPase, recent structural studies show that it is a component of the mature functioning mammalian V-ATPase^17,18^. The PRR was also identified for its ability to stimulate (pro) renin to actively cleave angiotensinogen^19^. Finally, the PRR was shown to be associated with Wnt signaling^20^. The function of PRR is associated with its cleavage by furin^21^. Intact V-ATPase isolated from mammalian brain was enriched in the transmembrane domain of the PRR^17,18^. Likewise the soluble extracellular domain released by furin has been thought to be the physiological stimulator (pro)renin^15^.

Based on the presence of full length PRR in osteoclast EVs, we hypothesized that the EVs from osteoclasts would be able to activate (pro)renin to cleave angiotensinogen. In the current study, we have tested this idea.

## Results

### EVs from RAW 264.7 osteoclast like cells and primary osteoclasts contain full length (pro) renin receptor and activate (pro)renin

**Figure 1:**
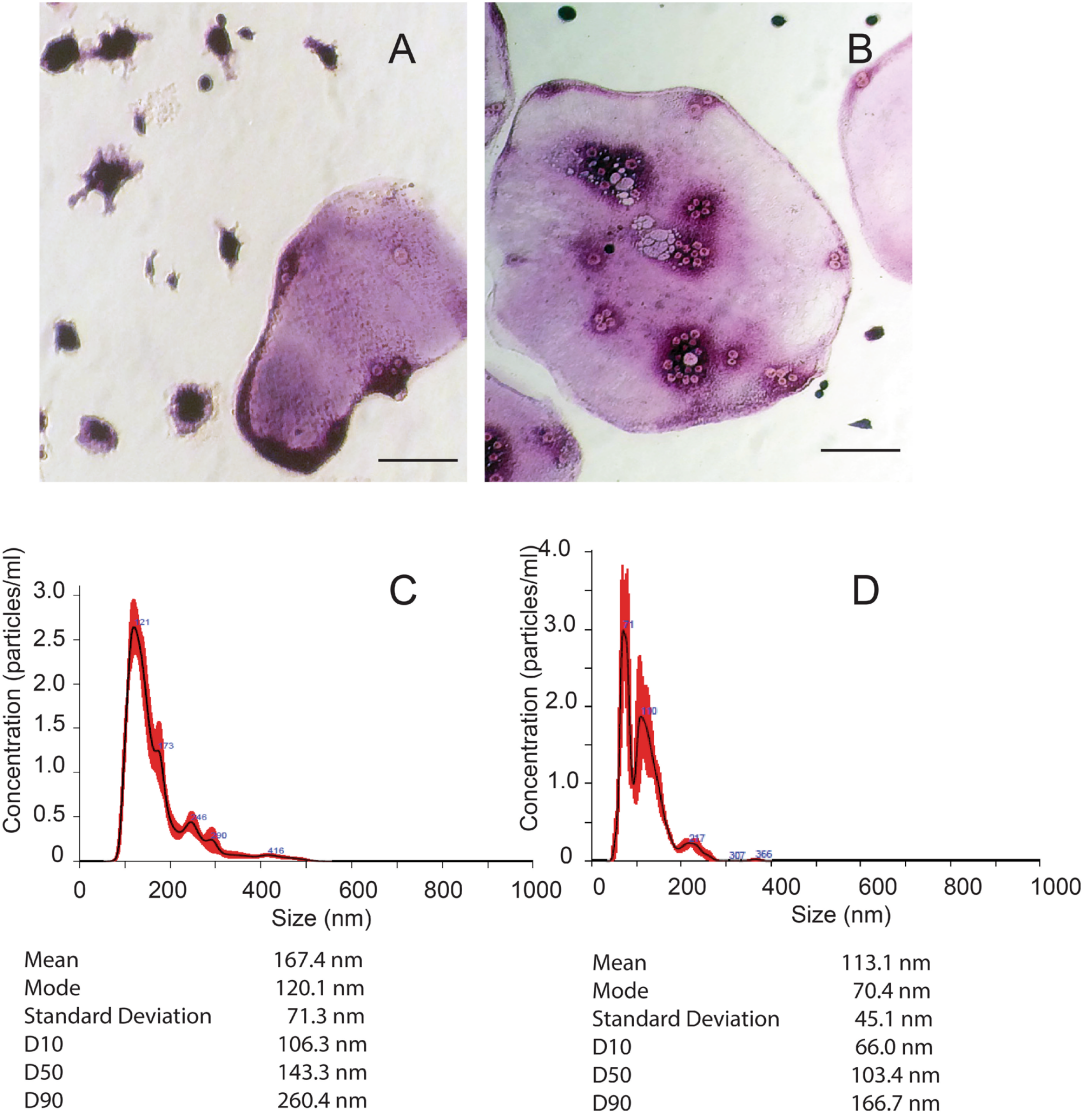
Characterization of EVs from RAW 264.7 osteoclast-like cells and primary mouse osteoclasts. RAW 264.7 cells or primary hematopoietic cells were stimulated to differentiate into osteoclasts. (**A**) RAW 264.7 osteoclast-like cells stained for TRAP activity (Pink is positive). Scale bar = 50 microns. (**B**) Primary osteoclasts stained for TRAP activity. Scale bar 50 microns. (**C**) Nanoparticle tracking of EVs collected from the conditioned media of RAW 264.7 osteoclast-like cells. (**D**) Nanoparticle tracking data of EVs collected from conditioned media of primary osteoclasts. The black track in the plots is the mean number for each size from 5 different 60 s trials, the red indicates the standard error boundaries at each size. The concentration of particles reflects the diluted concentration of the isolated EVs. Mean, and mode reflect the data from the particle size of the 5 trials. Standard Deviation reflects the spread of the data. D10, D50, and D90 indicates the size where 10, 50 or 90 percent of the detected particles are smaller, and reflects another measure of the spread of the data.

RAW 264.7 osteoclast-like cells and primary osteoclasts (Fig. 1A,B), were cultured and EVs were isolated. EVs were characterized by nanoparticle tracking (Fig. 1C,D) The sizes of EVs from RAW 264.7 osteoclast-like cells and primary osteoclasts were in the range of extracellular vesicles, but EVs from osteoclasts were smaller on average. This is consistent with the small size of EVs we have detected previously from primary osteoclasts.

**Figure 2:**
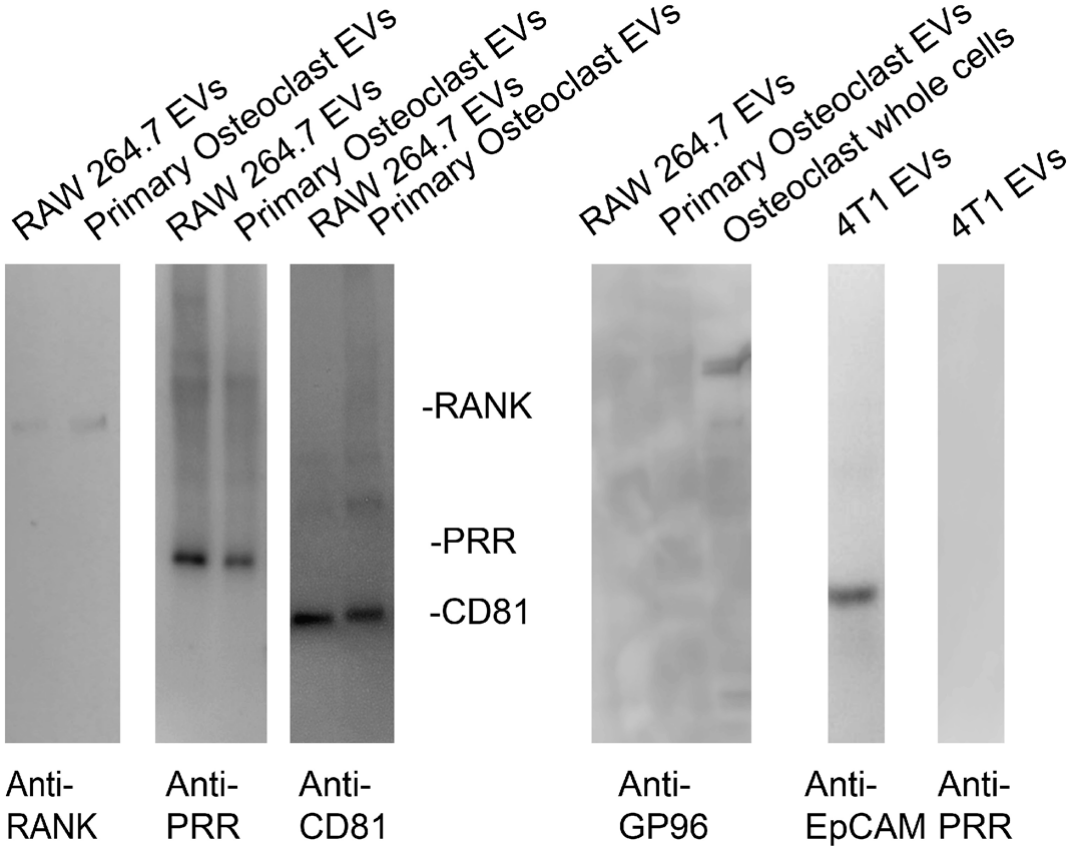
Immunoblots show that EVs isolated from RAW 264.7 osteoclast-like cells and primary osteoclasts have similar levels of RANK and PRR. First panel shows pre- stained molecular weight markers transferred to Immobilon P. The second panel is shows immunoblots of 5 × 10^8^ EVs isolated from conditioned media from RAW 264.7 osteoclast-like cells or primary osteoclasts stained with an anti-RANK antibody. The third panel shows staining the 5 × 10^8^ EVs with an anti-PRR antibody. The fourth panel shows staining of 5 × 10^8^ EVs with an anti-CD81 antibody. CD81 is a marker for EVs. The fifth panel shows an effort to stain 5 × 10^8^ EVs with an anti-GP96 antibody, which supports that the EVs are not contaminated with material from the endoplasmic reticulum, a common contaminant. A whole cell extract lane from osteoclasts is presented to show that the anti-GP96 antibody works. The sixth panel shows that 4T1 EVs (1 × 10^8^) have the expected protein marker EpCAM. The seventh panel demonstrates that we were unable to detect PRR in 1 × 10^8^ EVs isolated from conditioned media of 4T1 cells. As previously reported, these data show EVs from mouse osteoclasts and osteoclast-like cells contain RANK and full length PRR. They demonstrate that the amount of PRR in EVs from 4T1 cells is much lower.

Immunoblots for PRR and RANK confirmed their presence in EVs from RAW 264.7 osteoclast-like cells and primary osteoclasts (Fig. 2). As shown previously^13,14^, EVs from osteoclasts and osteoclast-like cells contained similar amounts of RANK. PRR was also detected in similar amounts and was 38 kD, consistent with the full-length protein. The tetraspanin CD81 was present as a positive control for EVs, while failure to detect the endoplasmic reticulum protein GP96 supports that there was little non-EV contamination. GP96 was detected in whole cell extracts of osteoclasts, while the PRR was not detected in EVs isolated from 4T1 cells (Fig. 2).

**Figure 3:**
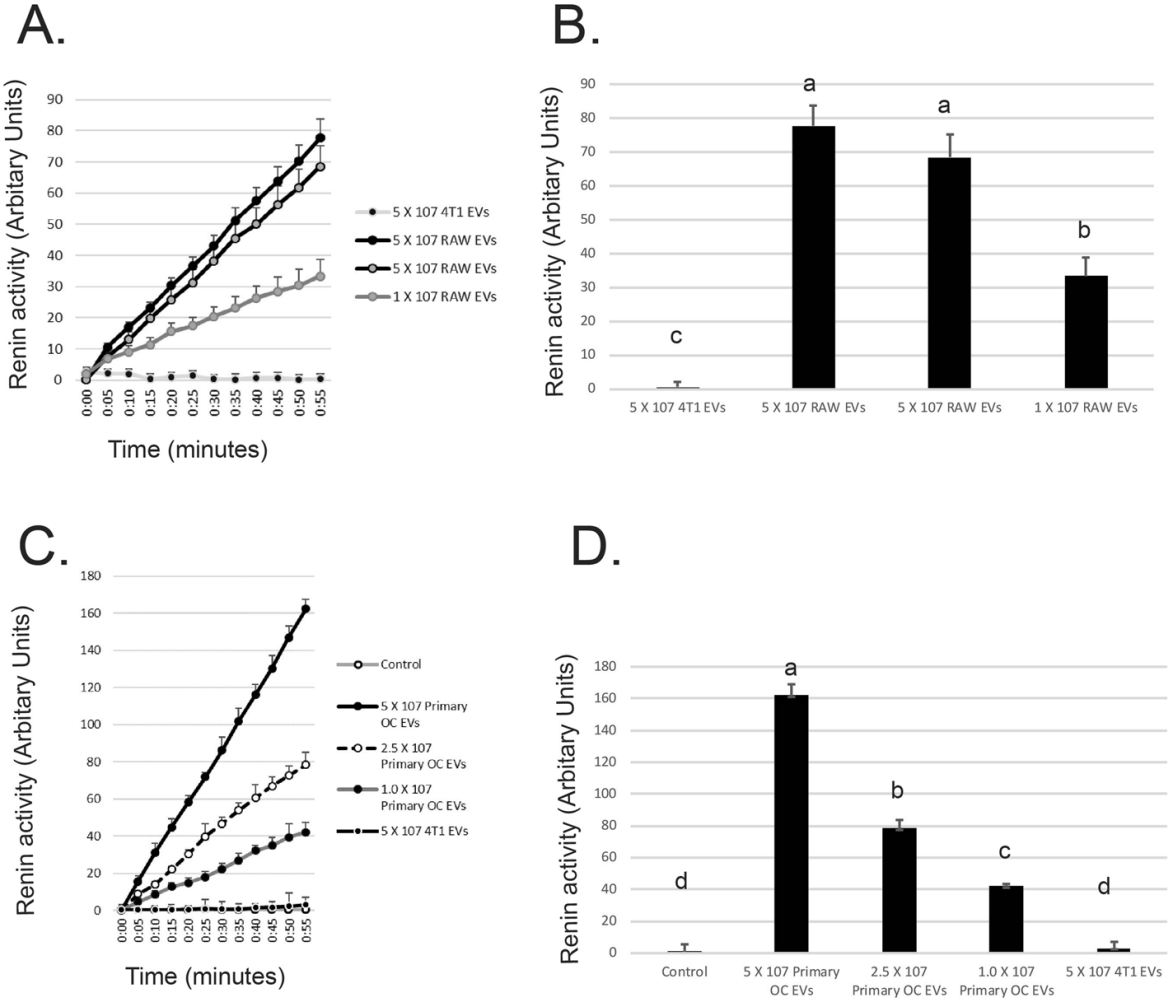
EVs from osteoclasts and osteoclast-like cells dose-dependently stimulate (Pro)renin to become active. (**A**) EVs from RAW 264.7 osteoclast-like cells stimulated (pro)renin to display renin activity. (**B**) At 55 min incubation, two independent batches of 5 × 10^7^ EVs from osteoclast-like cells stimulated (pro)renin in statistically different from 1 × 10^7^. EVs from osteoclast-like cells or 5 × 10^7^ EVs from 4T1 murine breast cancer cells used as a control. PRR is not detected in blots of 4T1 EVs. (**C**) EVs from primary osteoclasts stimulated (pro) renin to display renin activity. (**D**) At 55 min 5 × 10^7^, 2.5 × 10^7^ and 1 × 10^7^ EVs from primary osteoclasts dose-dependently stimulated (pro)renin. All were statistically-different from control (PBS) or a second control, 5 × 10^7^ 4T1 EVs. Letters indicate that group is different p < 0.05 by Student’s t test from groups with other letters within a figure.

EVs from osteoclasts and osteoclast-like cells dose–dependently stimulated (pro)renin to become proteolytically-active (Fig. 3A,B). Controls were EVs from 4T1 breast cancer cells, in which we were unable to detect PRR. EVs from primary mouse osteoclasts also dose-dependently stimulated (pro)renin activity (Fig. 3C,D). Controls included EVs from 4T1 cells and no EVs.

### The “handle region peptide” from (pro)renin blocks the ability of PRR in EVs to activate (pro)renin to cleave its substrate

**Figure 4:**
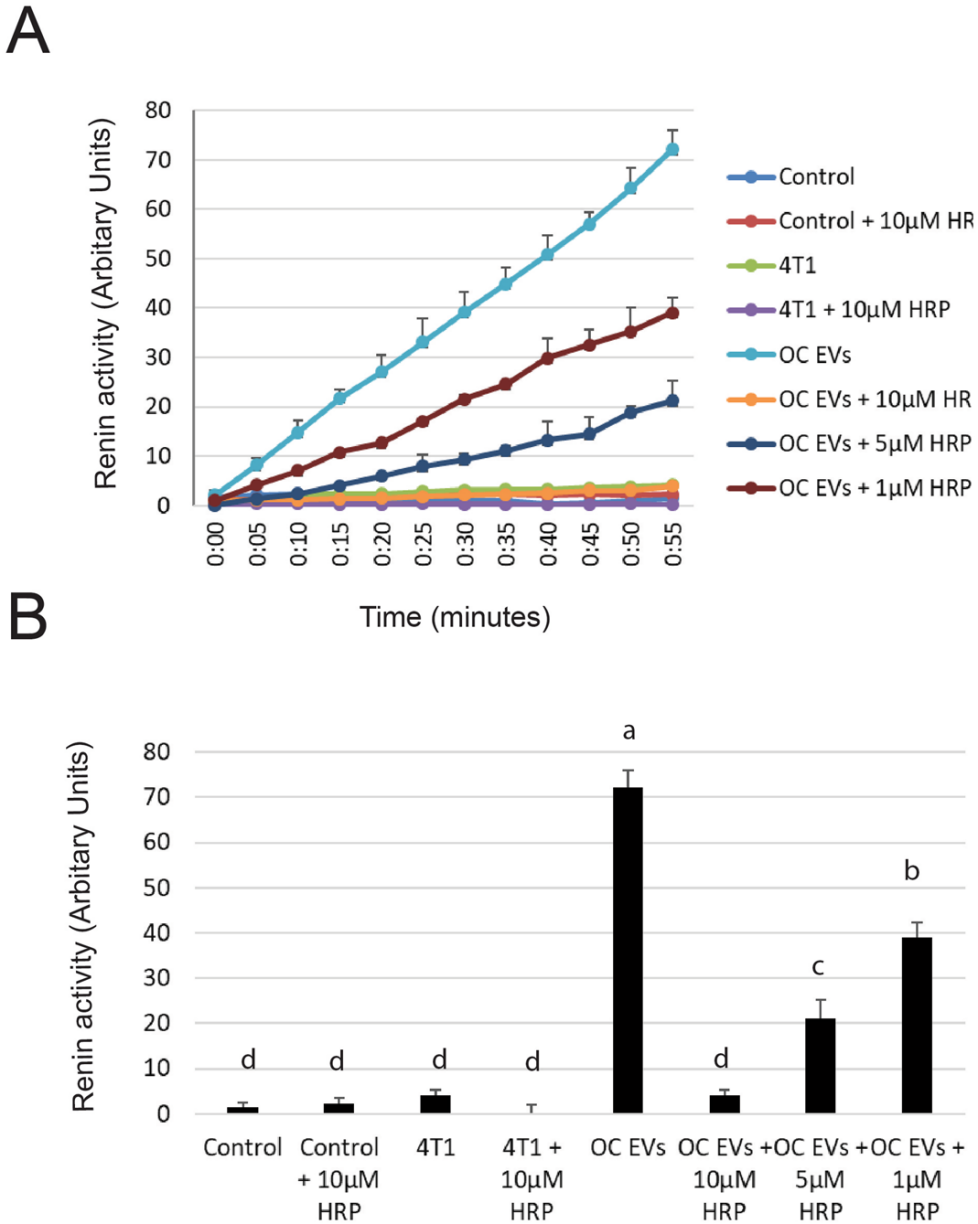
Handle Region Peptide (HRP) dose-dependently blocks activation of (pro)renin by EVs from osteoclasts. (**A**) EVs from osteoclasts (5 × 10^7^) stimulated (pro)renin activity, while activity was not stimulated by PBS control or EVs from 4T1 cells (5 × 10^7^). Addition of the HRP peptide dose-dependently reduced the stimulation of (pro)renin activity. (**B**) At 55 min, 1, 5 and 10 µM HRP reduced the ability of osteoclast EVs to stimulate (pro)renin activity in a statistically-significant manner. Letters indicate that group is different p < 0.05 by Student’s t test from groups with other letters within the figure.

The “handle region peptide” contains the sequence from a binding site in (pro)renin for the PRR, and has been shown to be a competitive inhibitor, blocking PRR’s ability to stimulate (pro)renin^22,23^. The handle region peptide dose-dependently blocked the ability of PRR-containing EVs to stimulate (pro)renin to be enzymatically active (Fig. 4A,B).

## Discussion

**Figure 5:**
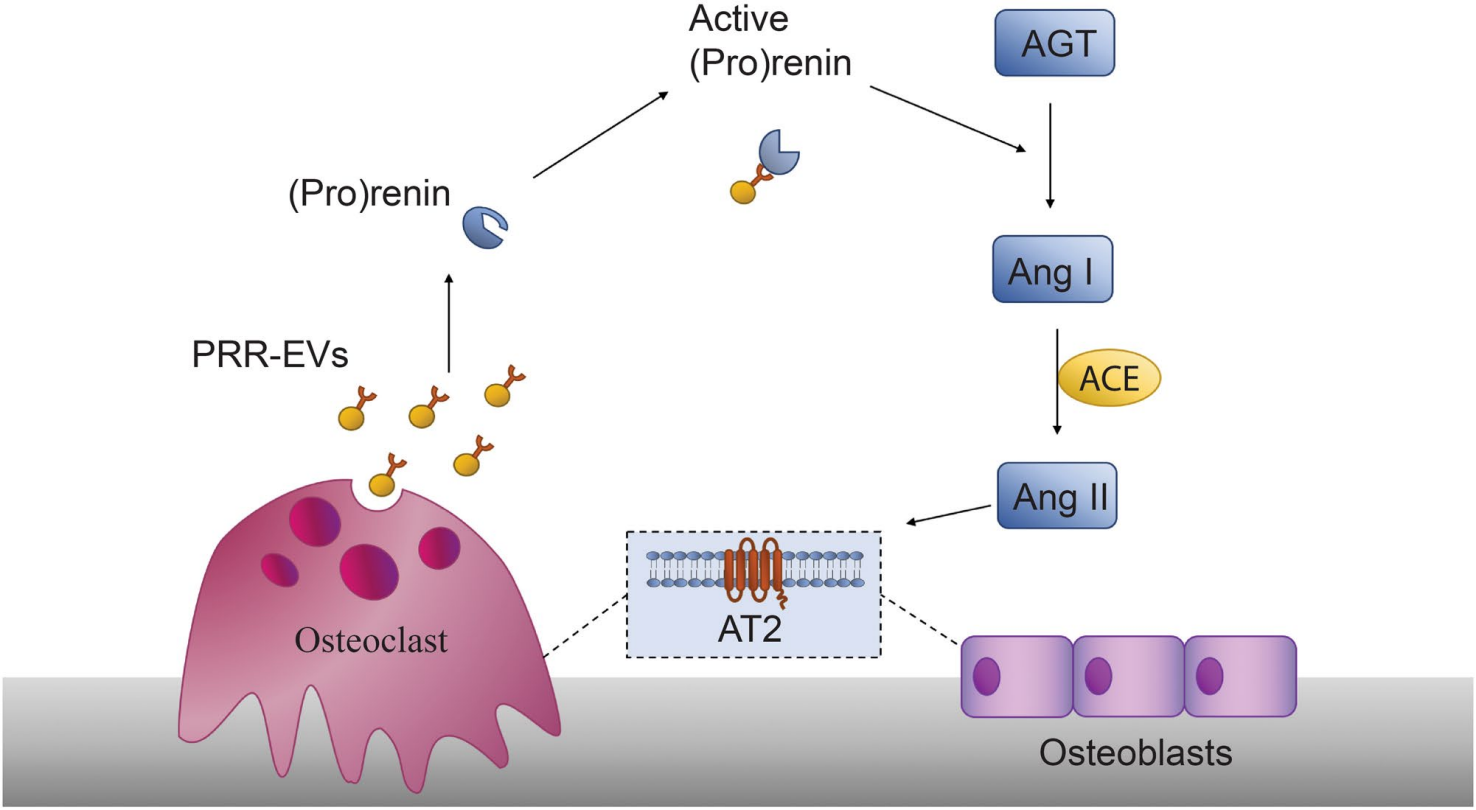
Schematic model of role of PRR-containing EVs shed by osteoclasts in local signaling in bone. EVs containing PRR (PRR EVs) are released in large numbers from osteoclasts. The PRR in the EVs binds (pro)renin eliciting a conformation change that activates it to cleave angiotensinogen (AGT) to angiotensin I (Ang I). Angiotensin converting enzyme (ACE) then cleaves Ang I to Ang II, which can bind angiotensin II (AT2) receptors on osteoclasts and osteoblasts. The net effect favors bone resorption in vivo although the mechanisms are still not well understood.

In this study, we identify a new potential regulatory mechanism associated with EVs, the ability of EVs that contain PRR to stimulate (pro)renin to become enzymatically active. Osteoclasts shed large amounts of PRR in EVs^11^, and stimulation of RAS is bone catabolic^7,8^. Both osteoclasts and osteoblasts have AT2 receptors and respond to RAS^24^. These data suggest that by stimulating RAS, EVs shed by osteoclasts may contribute to the stimulation of osteoclast activity directly (autocrine), or indirectly through osteoblasts (paracrine) (Fig. 5).

Three physiologic roles have been identified for the PRR: accessory protein/component of V-ATPase^16–18^, stimulator of (pro)renin^25,26^, and as a scaffold between V-ATPase and Frizzled, which stimulates Wnt/beta catenin signaling^20^. PRR is normally not present on the cell surface of most cells, and the ability to stimulate (pro)renin has been considered primarily a function of the cleaved extracellular domain. The fact that full length PRR, with the ability to stimulate (pro)renin, is present in EVs provides another route by which PRR can be exposed to the extracellular milieu, and potentially activate RAS.

The intact V-ATPase is composed of 16 subunits^17,18,27^. We reported that the PRR in osteoclast EVs was associated with a sub-complex of the V-ATPase^11^. This was intriguing since it is known that the PRR is required for V-ATPase assembly, although its exact role and mechanism in assembly are not known^15^. Very recent structural data show that membrane domains of PRR, along with a second accessory protein ATP6AP1/Ac45, are fully integrated into the functional mammalian V-ATPase from rat and bovine brains^17,18^. It is possible that the sub-complex found in osteoclast EVs represents a V-ATPase assembly intermediate that when exported in EVs gains a “moonlighting” function as a stimulator of RAS.

We used EVs from 4T1 breast cancer cells as a PRR-deficient control. We had originally expected that these cells, and their EVs, might be enriched in PRR and other V-ATPase subunits because plasma membrane V-ATPase has been shown to be involved in their metastatic activity^28^. However, when directly compared with osteoclasts the level of V-ATPase, and PRR is much lower in 4T1 cells compared with osteoclasts.

In summary, osteoclasts shed EVs that contain large amounts of uncleaved PRR. The PRR in the EVs stimulates (pro)renin to become active and thus would be expected to trigger increased local RAS, which favors bone resorption. Future studies will be necessary to confirm this idea. The ability to stimulate (pro)renin represents a new potential mechanism by which EVs can serve as intercellular regulators, and possibly, a new mechanism for controlling bone remodeling.

## Methods

### Reagents and antibodies

Dulbecco’s minimum essential media (dMEM) and minimum essential media, α modification (αMEM) were obtained from Sigma/Aldrich Chemical CO (St. Louis, MO). The anti-mouse PRR (10926-1-AP) was obtained from Thermo Fisher (Waltham, MA, USA). Anti-RANK antibody (Orb6560) was obtained from Biorbyt (Cambridge, UK). Anti-CD81 antibody (SC-166029) was obtained from Santa Cruz (Santa Cruz, CA). Anti-GP96 (36-2600) was obtained from Life Technologies (Carlsbad, CA, USA). The handle region peptide (RIPLKKMPSV) was synthesized by Biomatik (Cambridge, CA). Secondary antibodies were obtained from Sigma-Aldrich (St. Louis, MO). ExoQuick TC was obtained from Systems Biosciences (Mountain View, CA).

### Cell culture

Primary osteoclasts were grown from precursors obtained from mouse femora and tibiae as described^13^. The University of Florida Institutional Animal Care and Usage Committee approved all mouse protocols (University of Florida IACUC protocol number: 20180303097). The study adhered to the ARRIVE guidelines. All procedures were performed in accordance with guidelines of the National Institutes of Health of the United States. Mice (C57BL/6, Charles River) were sacrificed by cervical dislocation, bones were dissected, and marrow was flushed from the marrow space with α-MEM complete media (Sigma-Aldrich) plus 10% exosome-free fetal bovine serum (System Biosciences) with 1% L-glutamine (Thermo Fisher Scientific), and 1% penicillin/streptomycin/am using a syringe with a 25-gauge needle. Cells were seeded in T75 flasks at a concentration of 1.5 × 10^6^ cells/mL supplemented with 5 ng/mL recombinant murine Macrophage-Colony Stimulating Factor [CSF-1] (Peprotech, Rocky Hill, NJ, USA) and allowed to grow for 24 h at 37 °C and 5% CO2. Nonadherent cells were removed, and 5.9 × 10^5^ cells/mL adherent cells were seeded in 24-well plates or at 2.1 × 10^6^ on 6-well plates. All cultures were supplemented with 10 ng/mL CSF-1 and 5 ng/mL soluble recombinant RANKL (sRANKL)^29^ to stimulate differentiation of osteoclasts. To generate osteoclast-enriched cultures, cells were cultured for 5 or 6 days with α-MEM with 10% exosome free fetal bovine serum (System Biosciences) refreshed every 3 days.

4T1 murine breast cancer cells (kind gift of Gary Sahagian, Tufts University, Boston, MA) were grown in dMEM plus 10% exosome-free fetal bovine serum (Systems Biosciences)^30^. Conditioned media was collected while cells were 50—80% confluent.

### EV isolation

All steps in EV isolations were done under sterile conditions as described previously^11,13^. ExoQuick TC material from System Biosciences was used to isolate EVs from cultures of primary cells following the manufacturer’s instructions. The final pellet, containing EVs and ExoQuick, was diluted fivefold with phosphate-buffered saline (PBS) to induce the ExoQuick material to return to the soluble state. The samples were then spun at 200,000×*g* for 2 h in an Airfuge (Beckman Coulter, Brea, CA, USA) and the pellets were collected.

### Microscopy

Primary osteoclasts and RAW 264.7 osteoclast-like cells were fixed with 2% formaldehyde in PBS for 20 min, permeabilized with 0.5% Triton X-100 in PBS, then stained for tartrate resistant acid phosphatase (TRAP) activity using Leukocyte acid phosphatase kit (Sigma-Aldrich catalog #386A). Images were taken using a Nikon Diaphot phase contrast microscope (ELWD 0.3) at a magnification of 100 X.

### Nanoparticle tracking

The diameter size and concentration of EV population was determined using a NanoSight NS-300 (Malvern) as described previously^29^. Samples were evaluated using different dilutions in sterile-filtered PBS and videos recording for 60 s were used to estimate the concentration and size distribution of EVs by light scattering and Brownian motion. The Nanosight NTA Software analyzed raw data videos by triplicate.

### Western Blots

Protein samples were separated by sodium dodecyl sulfate polyacrylamide gel electrophoresis on 4–20% gels using the Mini-Protean system (BioRad). Gels were blotted to nitrocellulose or to Immobilon membranes (Pierce) and horse radish peroxidase-conjugated secondary antibodies were used to detect primary antibodies. These were detected either using a chromogenic substrate (ThermoFisher, CN/DAB substrate), or chemiluminescent substrate (ThermoFisher, Super Signal West Pico). Blots were either photographed or detected using a BioRad ChemiDoc MP (BioRad). The Optimal Autoexposure setting was used to acquire images. The Raw photographs and chemiluminescent data (included in Supplementary Fig. 1) were minimally processed using brightness and contrast controllers equally over the whole blot in Adobe Photoshop for final figures. All blots comply with the digital image and integrity policy.

### (Pro)renin activity assay

The SensoLyte 520 Mouse Renin Assay kit (Anaspec, Fremont, California) was used to assay for renin activity. This kit allows for continuous assay of renin activity using a 5-FAM/QXL 520 fluorescence resonance energy transfer (FRET) peptide. In the FRET peptide, the fluorescence of 5-FAM is quenched by QXL 520. Upon cleavage into two separate fragments by mouse renin, the fluorescence of 5-FAM is recovered. This was measured with a fluorescence multiwell plate reader (Synergy HTX, Biotek, Winooski, Vermont) at excitation/emission: 〖"λ " 〗_ex = 485 nm / 〖"λ " 〗_em = 528 nm. Fluorescence readings are expressed in relative fluorescence units (RFU). Instruments were calibrated using a 5-FAM fluorescence reference standard. The plates were placed in the multiwell plate reader for 60 min, with measurements taken every 5 min.

### Statistics

The results are expressed as mean plus/minus Standard Error. We used the program GraphPad Prism 5 (GraphPad Software, La Jolla, CA) to compare samples by One-Way ANOVA and Student’s t-test. P values < 0.05 were considered significant. Nanoparticle tracking was analyzed by ANOVA with Tukey’s modification^30^.

## Supplementary Information

Supplementary Figure 1.

## Data Availability

The datasets generated during and/or analyzed during the current study are available from the corresponding author on reasonable request.
