# Supplementary material for: Activation of (pro)renin by (pro)renin receptor in extracellular vesicles from osteoclasts
Source: Sci Rep. 2021 Apr 28;11:9214. doi: 10.1038/s41598-021-88665-y (PMC8080643; doi:10.1038/s41598-021-88665-y)
Supplement: Supplementary file 1 — Supplementary Figure 1. [file 41598_2021_88665_MOESM1_ESM.pdf]

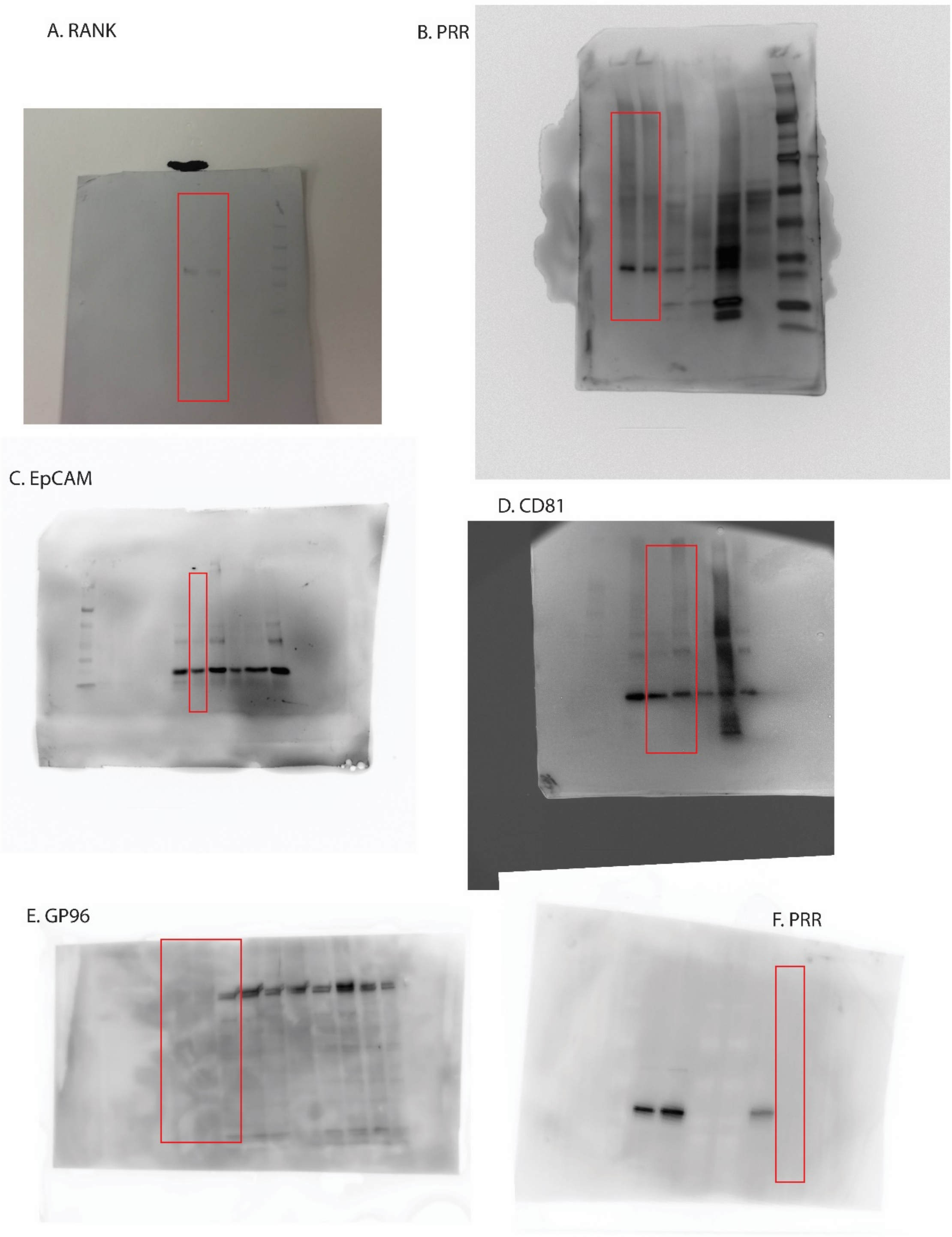

Supplementary Figure 1. Unprocessed images for Westerns shown in Figure 2. A. Photograph of colorimetrically- visualized blot of RANK. Red box indicates lanes in Figure 2. Other lanes on blot

include other samples. B. Chemiluminescent detection of PRR. Other lane are various cell extracts . C. Chemiluminescent detection of EpCAM. Additional lanes are other EV samples. D. Chemiluminescent-detection of CD81. Other lanes are different dilutions of the EVs samples shown and EV samples from other preps. The side by side are matched for EV numbers (by nanoparticle tracking). E. Chemiluminescent detection of GP96. Other lanes are cell extracts from RAW cells treated in various ways for another experiment. F. Chemiluminescent-detection of PRR. Positive lanes are EVs from RAW 264.7 cells, primary osteoclasts (left two lanes) and EVs from THP1 cells (lane next to 4T1 cells).
